# Genomic, morphological and functional characterisation of novel bacteriophage FNU1 capable of disrupting *Fusobacterium nucleatum* biofilms

**DOI:** 10.1038/s41598-019-45549-6

**Published:** 2019-06-24

**Authors:** Mwila Kabwe, Teagan L. Brown, Stuart Dashper, Lachlan Speirs, Heng Ku, Steve Petrovski, Hiu Tat Chan, Peter Lock, Joseph Tucci

**Affiliations:** 1 Department of Pharmacy and Biomedical Sciences, La Trobe Institute for Molecular Science, La Trobe University, Victoria, Australia; 2 Melbourne Dental School, University of Melbourne, Victoria, Australia; 3 Department of Physiology, Anatomy and Microbiology, La Trobe University, Victoria, Australia; 4 Department of Microbiology, Royal Melbourne Hospital, Victoria, Australia; 5 La Trobe Institute for Molecular Science, La Trobe University, Victoria, Australia

**Keywords:** Colon cancer, Biofilms, Infection control in dentistry, Bacteriophages, Applied microbiology

## Abstract

*Fusobacterium nucleatum* is an important oral bacterium that has been linked to the development of chronic diseases such as periodontitis and colorectal cancer. In periodontal disease, *F. nucleatum* forms the backbone of the polymicrobial biofilm and in colorectal cancer is implicated in aetiology, metastasis and chemotherapy resistance. The control of this bacteria may be important in assisting treatment of these diseases. With increased rates of antibiotic resistance globally, there is need for development of alternatives such as bacteriophages, which may complement existing therapies. Here we describe the morphology, genomics and functional characteristics of FNU1, a novel bacteriophage lytic against *F. nucleatum*. Transmission electron microscopy revealed FNU1 to be a large *Siphoviridae* virus with capsid diameter of 88 nm and tail of approximately 310 nm in length. Its genome was 130914 bp, with six tRNAs, and 8% of its ORFs encoding putative defence genes. FNU1 was able to kill cells within and significantly reduce *F. nucleatum* biofilm mass. The identification and characterisation of this bacteriophage will enable new possibilities for the treatment and prevention of *F. nucleatum* associated diseases to be explored.

## Introduction

*Fusobacterium nucleatum* is a Gram-negative facultative anaerobic bacillus that is a normal component of the oral microbiome. It has been associated with periodontal diseases^1^ as well as malignancies of the oral cavity, head and neck, oesophagus, cervix, stomach and colon^2–4^. This association with a range of malignancies has led to its referral as an “oncobacterium”^4^. In these diseases, *F. nucleatum* biofilms have been demonstrated to play a critical role.

Chronic periodontitis results from a dysbiosis in subgingival plaque biofilm communities that leads to the emergence of pathogenic species that dysregulate the host immune response leading to sustained and uncontrolled inflammation^5,6^. In chronic periodontitis, *F. nucleatum* has been shown to act as a backbone for pathogenic subgingival polymicrobial biofilms by forming a bridge between the more commensal early colonisers and the more pathogenic late colonisers^1,7,8^. This microbial biofilm is therefore responsible for the initiation and progression of chronic periodontitis^9,10^. Apart from their role in periodontitis, bacterial biofilms and microbiota organisation have also been associated with gut tumours^11^. In colorectal cancer, *Fusobacterium* has been demonstrated to be intimately involved in modulating the tumour immune microenvironment and recruiting myeloid cells that assist in tumorigenesis, tumour cell proliferation and metastasis^12^, modulating autophagy and resistance to chemotherapies^13^.

Current treatment of periodontal disease is mechanical debridement with antibiotics and antiseptics as adjuncts. However, this approach is not without controversy^14^, with the development of antibiotic resistance being a major caveat^15^, along with dysbiosis of oral microbiota contributing to inflammation and disease recurrence^16,17^. Current treatment of colorectal cancer includes surgery, chemotherapy and radiation therapy. In both cases, a targeted therapy that specifically attacks *Fusobacterium* in biofilms can potentially provide a new modality to combat these important diseases. Optimal treatment of biofilms in periodontitis and colorectal cancer would target specific bacteria in the biofilms, have minimal inflammatory effect, and a low risk for resistance development by bacteria.

Alternatives to antibiotics that have a narrow range in their bacterial targets, and capable of breaking down bacterial biofilms are bacteriophages^18,19^. Bacteriophages, which can be either lytic or temperate^20^, have been involved in an evolutionary arms race with bacteria, and as such are capable of overcoming or adapting to development of bacterial resistance against them^21^. Temperate bacteriophages are present in bacteria in a latent phase until certain conditions leading to bacterial cellular damage occurs e.g. exposure to ultra-violet radiation. Lytic bacteriophages, on the other hand, will lyse bacteria after infection and have potential for therapeutic use^20^. *F. nucleatum* is an important micro-organism in the structural composition of biofilms in periodontitis and colon cancer and as such provides a useful target for bacteriophages. To date, however, only temperate bacteriophages of *F. nucleatum*, ɸFunu1 and ɸFunu2, have been fully characterised^22^. There has been a single report of a lytic bacteriophage against *F. nucleatum*, Fnpɸ02 that has been isolated and phenotypically characterised^23^. Full genomic characterisation was not performed, and short amplicons within Fnpɸ02 showed homology ranging from 84% to 98% to *Cutibacterium acnes* (formally *Propionibacterium acnes*) bacteriophages. The genes where these amplicons were taken are highly conserved in *C. acnes* bacteriophages. *C. acnes* has been isolated frequently with *F. nucleatum*^24^ but no bacteriophages have been found that target both *F. nucleatum* and *C. acnes*.

Bacteriophages lytic against other types of bacteria such as *Pseudomonas aeruginosa*^25^, *Escherichia coli*^26^ and *Streptococcus mutans*^27^ have been shown to be able to disrupt mono-biofilms formed by their respective hosts. The potential exists for use of bacteriophages against biofilms in periodontal infection^18,19^ and colon cancer. We describe here the full genomic and morphological characterisation of a novel lytic bacteriophage, FNU1, which is capable of disrupting existing *F. nucleatum* biofilms. This bacteriophage has a novel genome (NCBI Genbank Accession Number: MK554696), with little homology to other viruses, and offers the potential for development to prevent and treat *F. nucleatum*-associated oral disease and cancers.

## Methods

### Ethics

All methods were performed in accordance with the La Trobe University Ethics, Biosafety and Integrity guidelines and regulations. Informed consent was obtained from participants for their involvement and use of samples in this study. The study protocols were approved by the La Trobe University Ethics Committee, reference number: S17-112.

### *Fusobacterium nucleatum* bacterial growth conditions

*Fusobacterium nucleatum* (ATCC 10953) that had been completely characterised^28^ and previously studied in a polymicrobial biofilm^29^ was used for all experiments. The cultures were grown in brain heart infusion media (BHI; Oxoid, Australia) supplemented with 0.5% cysteine (Sigma, Australia) and 0.5% haemin (Sigma, Australia) in either broth or agar. The cultures were grown anaerobically using anaerobic generating packs (AnaeroGen) (Oxoid, Australia) at 37 °C. For this study, the identity of the strain was confirmed using 16 S rRNA gene amplification and sequencing via U27F: 5′AGAGTTTGATCMTGGCTCAG3′ and U1492R: 5′AAGGAGGTGWTCCARCC 3′ primers^30^. The thermocycling conditions were 95 °C for 3 minutes, 32 cycles of 95 °C for 30 seconds, 60 °C for 30 seconds, and 72 °C for 90 seconds, with a final extension at 72 °C for 10 minutes. The amplicons were cleaned using QIAquick^®^ PCR purification kits (Qiagen, Australia) and analysed by Sanger sequencing at the Australian Genome Research Facility (AGRF) in Queensland, Australia.

### Bacteriophage isolation

Mouthwash samples were collected from dental practices in Bendigo (Victoria, Australia) and screened for the presence of lytic bacteriophages against *F. nucleatum* using the enrichment method in BHI broth according to Gill and Hyman^31^. Briefly, 100 µL of *F. nucleatum* grown previously in broth culture for 48 hours anaerobically was added to 1 mL of sample and 20 mL of broth and incubated anaerobically at 37 °C for seven days. The enrichment was filtered using a 0.20 µm cellulose acetate filter (Microanalytix, Australia) before spotting 10 µL of the filtrate on a freshly prepared lawn of *F. nucleatum* on 1% agar. The plate was incubated for 48 hours anaerobically at 37 °C. Observed plaques were excised and purified as described previously^32^. To test the host range of the purified bacteriophage, it was also spotted onto cultures of *Streptococcus mutans, Porphyromonas gingivalis* and *C. acnes*, which are all found in the oral cavity.

### Electron microscopy

The purified bacteriophage particles were visualised using a JEOL JEM-2100 Transmission Electron Microscope (TEM) using 400-mesh carbon-coated copper grids (ProSciTech, Australia). The bacteriophage lysate was allowed to adsorb to the grid for 30 seconds before being washed with Milli-Q® water (Promega, Australia). The adsorbed particles were then negatively stained twice for 30 seconds with 2% [W/V] uranyl acetate (Sigma, Australia). Excess stain on the grids was removed using filter paper before being air dried for 20 minutes. The grid was visualised and images captured with a Gatan Orius SC200D 1 wide-angle camera (Gatan Microscopy Suite and Digital Micrograph Imaging software version 2.3.2.888.0) at 200 kV. Further image analysis was achieved in ImageJ software version 1.8.0_112.

### DNA extraction

Bacteriophage DNA was extracted from a highly concentrated purified lysate (approximately 10^11^ PFU mL^−1^) using the phenol-chloroform method as previously described^32^. All compounds used in the DNA extraction process were obtained from Sigma-Aldrich (Australia) unless stated otherwise. The concentrated bacteriophage stock was treated with 5 mmol L^−1^ of MgCl_2_ and 1.0 µL each of RNase A (Promega, Australia) and DNase I (Promega, Australia) to a final concentration of 10 µg mL^−1^. The solution was incubated at room temperature for 30 minutes to digest extraneous DNA or RNA. Polyethylene glycol 8000 (PEG) at 10% [W/V] and sodium chloride (NaCl at 1 g L^−1^) were added to the mixture and incubated overnight. The solution was centrifuged at 12000 × *g* for 5 minutes and pellets resuspended in 50 µL nuclease-free water (Promega, Australia). Bacteriophage proteins were digested by the addition of Proteinase K (50 µg mL^−1^), EDTA (20 mmol L^−1^) and sodium dodecyl sulphate (0.5% (v/v)) and incubating for one hour at 55 °C. An equal volume of phenol-chloroform-isoamyl alcohol (29:28:1) was then added to separate viral DNA from proteins. The mixture was gently vortexed and then centrifuged at 12000 × *g* for 10 minutes to isolate the aqueous phase. DNA was precipitated by adding an equal volume of isopropanol and incubating overnight at −20 °C. The DNA pellet was collected by centrifugation at 12000 × *g* for 5 minutes. The DNA pellet was washed in 70% ethanol, air-dried and finally resuspended in 30 µL of nuclease-free water (Promega, Australia).

### Whole genome sequencing and *in-silico* analysis

Nextera® XT DNA sample preparation kits were used to prepare DNA libraries according to the manufacturer’s instructions (Illumina, Australia). The libraries were sequenced on an Illumina MiSeq® using a MiSeq® V2 reagent kit (300 cycles) with 150 basepair (bp) paired end reads. Sequence reads were assembled *de novo* using Geneious software version 11.0.5. Gene prediction was achieved by predicting open reading frames (ORFs) using ATG, GTG, and TTG start codons with a minimum nucleotide length of 50 bp. The ORFs were translated using Geneious and analysed by BLASTP (https://blast.ncbi.nlm.nih.gov/) to ascribe potential function. The genome was further examined for the presence of transfer RNA (tRNA) and transfer-messenger RNA (tmRNA) using ARAGORN^33^ and tRNAscan-SE 2.0^34^. Whole genome alignments and phylogenetic tree construction were performed in CLC genomics workbench version 9.5.4 by UPGMA algorithm with 1,000 replicate bootstrapping. The alignment included whole genomes of FNU1 and other bacteriophages specific for oral bacteria obtained from the NCBI Genbank.

### Biofilm growth and quantification

Biofilm experiments were conducted anaerobically using BHI broth (Oxoid, Australia) supplemented with 0.5% cysteine, 0.5% haemin and 0.5% glucose in 96 well polystyrene plates (Greiner bio-one, Australia) coated with 0.5% gelatin. *F. nucleatum* was cultured for 48 hours and 100 µL of approximately 1 × 10^8^ CFU mL^−1^ of *F. nucleatum* in exponential growth phase was added to each well before an equal volume of broth was added. The inoculated plates were incubated at 37 °C under anaerobic conditions and shaking at 120 rpm (Ratek Medium Orbital shaking incubator) for 4 days with sterile broth replenishment after 48 hours. To each well, 10 µL of bacteriophage FNU1 at 10^11^ PFU/mL was added after 4 days of biofilm formation. The biofilms were then incubated for a further 24 hours anaerobically at 37 °C before quantification assays completed as described previously^35^. Briefly, planktonic cells were washed off gently using MilliQ® deionised water (Merck, Australia) and the attached cells air dried. The attached biomass was then stained with 200 µL of 0.1% crystal violet for 5 minutes, washed using deionised water and air-dried for 5 minutes. An equal volume of ethanol (70%) was added to each well to decolourise the stained attached cells and the absorbance of the crystal-violet stained ethanol was evaluated at a wavelength of 600 nm (OD_600_) using a FlexStation 3 plate reader (Molecular Devices, United States).

### Biofilm viability analysis

To test for viability, the *F. nucleatum* biofilm was grown on gelatin-coated microscope slides in the same manner as in the 96 well plates described above. SYBR gold® and Propidium Iodide (PI) were used to stain nucleic acids of live (membrane intact) and dead (membrane compromised) cells, respectively. Propidium Iodide (3 µL) was added to 100 µL of SYBR gold ® diluted (1:100) in dimethyl sulfoxide (Sigma, Australia). The mixture was applied to the biofilm on slides and incubated for 30 minutes. Excess PI and SYBR gold ® were rinsed off and slides air-dried before mounting with 5 µL Vectorshield® (Burlingame, USA) and coverslips. The slides were examined using an Olympus Fluoview Fv10i-confocal laser-scanning microscope (Olympus Life Science, Australia) with excitation wavelength at 485 nm. Green emission at fluorescence 535 nm and Red emission at fluorescence 635 nm were measured to indicate live versus dead cells on the slides.

### Statistical analysis

The absorbance values quantifying the biofilms were analysed for normality using the Shapiro Wilk test and the medians compared by a paired T-test. The p-values of less than 0.05 were considered statistically significant. All statistical analysis was performed using the Statistical Package for Social Sciences (SPSS version 25).

## Results

### Isolation and phenotypic characterisation of *F. nucleatum* bacteriophage FNU1

**Figure 1:**
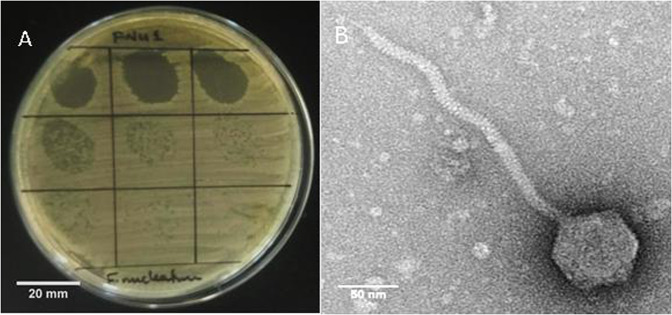
(**A**) Bacteriophage FNU1 spotted onto *F. nucleatum* culture on BHI with 1% agar. From the top left to the bottom right, each square represents a 10-fold serial dilution of FNU1. Clearing is seen at the highest concentrations with individual plaques discernible at subsequent dilutions. Each single plaque is ≈1 mm diameter. (**B**) TEM image of FNU1 revealing *Siphoviridae* bacteriophage with long ≈310 nm tail and icosahedral head diameter of ≈88 nm.

One mouthwash sample was found to produce clear plaques on 1% BHI agar. This was not observed on BHI with 1.5% agar. On the less concentrated 1% agar, clear round plaques of approximately 1 mm diameter were seen (Fig. 1A). The host range of FNU1 was restricted to *F. nucleatum*, and did not extend to other bacteria found in the oral cavity that we tested. TEM revealed a *Siphoviridae* bacteriophage with an icosahedral head of ≈88 nm in diameter and a long flexible tail terminating in a spike (Fig. 1B). The tail was approximately 310 nm long and ≈10 nm wide with a spike at the end, measuring an average of ≈20 nm long and ≈5 nm wide on the widest section.

### Genomic analysis

Bacteriophage DNA extraction and sequencing was performed on three separate occasions. On all occasions, a single contig of 130914 bp was obtained with coverage ranging from 121 to 2400 times. The process was repeated to ensure accuracy and because the sequence generated displayed low homology to any known bacteriophage or other genomes present in the database. The FNU1 bacteriophage genome (NCBI Genbank Accession Number: MK554696) was composed of 178 predicted ORFs of which 30.34% (54/178) had no significant homology to any sequences in the Genbank database and 38.20% (68/178) had some similarity but with E values of less than 1e-4. Of the remaining genome for bacteriophage FNU1, 31.46% (56/178 ORFs) had significant homology to other sequences, and of these, 71.43% (40/56 ORFs) had conserved domains. The overall GC content was 25.0%. The ORFs, their significant matches and E values are shown in Table 1.

**Table 1:** Putative FNU1 proteins and their homology to published sequences.

ORF	Coordinates	Size (aa)	Homology to known sequences in NCBI database	% Identity	% Query cover	E0 value
ORF1	1..1659	553	Gifsy-2 prophage tail fiber protein, partial [*Salmonella enterica* subsp. *enterica* serovar Newport str. SHSN010]	55	9	1.00E − 06
ORF2	1770..2267	166	No significant similarity found			
ORF3	2264..2464	67	No significant similarity found			
ORF4	2457..2855	133	Hybrid sensor histidine kinase/response regulator [*Pedobacter heparinus*]	47	28	9.40E + 00
ORF5	2857..3399	181	No significant similarity found			
ORF6	3396..4223	276	Nucleotidyltransferase domain-containing protein [*Balneola* sp.]	35	92	2.00E − 29
ORF7	4217..4948	244	Hypothetical protein [*Bacillus* sp.]	35	97	2.00E − 35
ORF8	4950..5366	139	ComF family protein [*Yoonia litorea*]	31	38	8.30E − 01
ORF9	5575..5796	74	rfaE bifunctional protein [*Desulfurobacterium thermolithotrophum* DSM 11699]	27	60	7.60E − 01
ORF10	5884..6558	225	Hypothetical protein [*Staphylococcus xylosus*]	40	91	2.00E − 41
ORF11	6577..7395	273	Antirepressor [*Fusobacterium necrophorum*]	39	97	7.00E − 44
ORF12	8220..8831	204	Hypothetical protein [*Olleya sp*. VCSM12]	30	58	8.40E + 00
ORF13	8835..9557	241	No significant similarity found			
ORF14	9557..11392	612	Hypothetical protein DRJ01_10315, partial [*Bacteroidetes bacterium*]	28	77	5.00E − 41
ORF15	11402..12949	516	Hypothetical protein DRJ01_10320 [*Bacteroidetes bacterium*]	29	96	1.00E − 41
ORF16	13878..14297	140	Methyl-CpG-binding domain-containing protein 9 [*Cicer arietinum*]	27	73	7.40E + 00
ORF17	14397..15473	359	No significant similarity found			
ORF18	15775..16071	99	No significant similarity found			
ORF19	16073..16444	124	Myosin-IB-like [*Plutella xylostella*]	31	50	8.50E + 00
ORF20	16437..16991	185	Hypothetical protein UU59_C0024G0011 [candidate division WWE3 bacterium GW2011_GWE1_41_27]	26	89	1.00E + 00
ORF21	16988..17623	212	No significant similarity found			
ORF22	17665..18798	378	Type I secretion system permease/ATPase [*Mesorhizobium sp*. WSM4313]	44	16	3.30E + 00
ORF23	18866..19642	259	No significant similarity found			
ORF24	19639..20283	215	No significant similarity found			
ORF25	20293..26217	1975	Phage tail tape measure protein, TP901 family, core region [*Cetobacterium ceti*]	24	26	4.00E − 20
ORF26	26279..27295	339	Hypothetical protein [*Brevibacillus fluminis*]	43	12	7.90E + 00
ORF27	27390..28244	285	Hypothetical protein [*Fusobacterium periodonticum*]	42	70	4.00E − 31
ORF28	28300..28674	125	No significant similarity found			
ORF29	29187..32576	1130	DUF1983 domain-containing protein [*Rhizobium subbaraonis*]	26	13	3.00E − 05
ORF30	33217..34371	385	Transposase [*Fusobacterium sp*. CM1]	84	97	0.00E + 00
ORF31	34498..35637	380	Hypothetical protein [*Cetobacterium sp*. ZWU0022]	41	43	1.00E − 26
ORF32	35640..36641	334	No significant similarity found			
ORF33	36653..37438	262	Hypothetical protein AKJ51_02780 [candidate divison MSBL1 archaeon SCGC-AAA382A20]	25	29	2.70E + 00
ORF34	37551..38327	259	ATP/GTP-binding protein [*Streptococcus parasanguinis*]	37	96	5.00E − 26
ORF35	38303..38713	137	No significant similarity found			
ORF36	39575..39889	105	Anaerobic ribonucleoside-triphosphate reductase [*Fusobacterium perfoetens*]	44	90	3.00E − 18
ORF37	41026..41247	74	No significant similarity found			
ORF38	(41608..41883)	92	No significant similarity found			
ORF39	(42013..42279)	89	Homoserine kinase [*Fusobacterium* sp. CM1]	47	47	8.60E + 00
ORF40	(42455..42775)	107	No significant similarity found			
ORF41	(43304..43540)	79	No significant similarity found			
ORF42	(44438..44680)	81	Serine–tRNA ligase [*Candidatus Daviesbacteria* bacterium RIFCSPHIGHO2_01_FULL_37_27]	40	53	7.40E + 00
ORF43	(44816..45196)	127	Hypothetical protein [*Lactobacillus reuteri*]	48	52	2.00E − 11
ORF44	(45315..45611)	99	Hypothetical protein [uncultured Mediterranean phage uvMED]	42	65	9.00E − 03
ORF45	(46128..46544)	139	No significant similarity found			
ORF46	(46574..46885)	104	No significant similarity found			
ORF47	(46903..47127)	75	Hypothetical protein J132_07476 [*Termitomyces* sp. J132]	37	65	6.20E + 00
ORF48	(47138..47506)	123	Alkaline phosphatase [*Stackebrandtia nassauensis*]	23	75	9.20E + 00
ORF49	(47521..47745)	75	No significant similarity found			
ORF50	(48158..48322)	54	No significant similarity found			
ORF51	(48727..49209)	161	Anaerobic ribonucleoside-triphosphate reductase activating protein [*Fusobacterium* sp.]	51	91	2.00E − 50
ORF52	(49458..49658)	67	No significant similarity found.			
ORF53	(49670..49996)	108	Hypothetical protein DV735_g31 [*Chaetothyriales* sp. CBS 134920]	36	71	7.50E − 01
ORF54	(50006..50467)	154	No significant similarity found			
ORF55	(50467..50955)	163	No significant similarity found			
ORF56	(51137..52471)	445	Anaerobic ribonucleoside-triphosphate reductase [*Fusobacterium perfoetens*]	53	98	3.00E − 167
ORF57	(52800..53834)	345	GIY-YIG nuclease family protein [*Clostridioides difficile*]	42	55	1.00E − 35
ORF58	(53905..54813)	303	Anaerobic ribonucleoside-triphosphate reductase [*Fusobacterium varium*]	46	98	1.00E − 71
ORF59	(54800.55060)	87	Hypothetical protein OFPII_09960 [*Osedax symbiont* Rs1]	38	51	8.30E − 01
ORF60	(55064..55387)	108	No significant similarity found			
ORF61	(55384..55851)	156	No significant similarity found			
ORF62	(56004..56456)	151	No significant similarity found			
ORF63	(56485..56724)	80	Mitochondrial carrier homolog 2 [*Drosophila arizonae*]	40	53	8.50E + 00
ORF64	(56733..56978)	82	Glycosyltransferase [*Algoriphagus resistens*]	36	59	8.10E − 01
ORF65	(56985..57296)	104	No significant similarity found			
ORF66	(57298..57597)	100	No significant similarity found			
ORF67	(58609..58989)	127	Outer membrane protein assembly factor BamA [*Sutterella parvirubra*]	27	62	3.40E + 00
ORF68	(59003..59752)	250	Hypothetical protein [*Fusobacterium nucleatum*]	33	31	1.70E − 02
ORF69	(59754..60326)	191	Hypothetical protein [*Fusobacterium periodonticum*]	37	32	8.60E − 01
ORF70	(60323..60697)	125	No significant similarity found			
ORF71	(60676..60894)	73	ATP-dependent DNA ligase [*Rhizophagus irregularis*]	37	67	5.90E + 00
ORF72	(60916..61179)	88	Hypothetical protein [*Desulfobacula toluolica*]	26	70	5.60E − 01
ORF73	(61184..61369)	62	No significant similarity found			
ORF74	(61359..61799)	147	Hypothetical protein SAMN05444672_10818 [*Bacillus* sp. OK838]	50	28	2.40E + 00
ORF75	(61796..62185)	62	No significant similarity found			
ORF76	(62176..62544)	123	No significant similarity found			
ORF77	(62560..63000)	147	No significant similarity found			
ORF78	(63013..63480)	156	Molecular chaperone DnaJ [*Paenibacillus* sp. Soil724D2]	32	91	2.00E − 19
ORF79	(63778..64101)	108	DUF1874 domain-containing protein [*Defluviitalea phaphyphila*]	57	97	2.00E − 31
ORF80	(64133..64306)	58	Hypothetical protein HMPREF1127_1046 [*Fusobacterium necrophorum subsp. funduliforme* Fnf 1007]	49	82	1.00E − 07
ORF81	(64450..64671)	74	DNA segregation ATPase FtsK/SpoIIIE, S-DNA-T family [*Crenotalea thermophila*]	45	56	7.10E + 00
ORF82	(64738..65517)	260	Radical SAM protein [*Clostridium botulinum*]	49	94	5.00E − 79
ORF83	(65598..68396)	933	Hypothetical protein [*Fusobacterium necrophorum*]	36	6	8.80E − 01
ORF84	(68490..68984)	165	Hypothetical protein [*Fusobacterium hwasookii*]	41	88	6.00E − 32
ORF85	(68984..69172)	63	Hypothetical protein [*Fusobacterium periodonticum*]	68	98	3.00E − 22
ORF86	(69291..69605)	105	Hypothetical protein YYE_03786 [*Plasmodium vinckei vinckei*]	36	62	5.20E − 01
ORF87	(69589..69942)	118	Na + /H + antiporter [*Porphyromonas* sp. oral taxon 279]	26	73	3.40E + 00
ORF88	(69911..70651)	247	No significant similarity found			
ORF89	(70663..71040)	126	Hypothetical protein [*Lactobacillus* sp. CBA3605]	33	41	5.30E + 00
ORF90	(71042..71473)	144	Guanylate-binding protein 6-like [*Eptesicus fuscus*]	37	48	2.90E − 01
ORF91	(71466..71660)	65	Hypothetical protein [*Rhodopirellula* sp. SWK7]	43	64	1.40E + 00
ORF92	(71647..71922)	92	Tyrosine recombinase XerC [*Chloracidobacterium thermophilum*]	41	47	8.60E + 00
ORF93	(72047..72946)	300	No significant similarity found			
ORF94	(73058..73651)	198	Hypothetical protein CTER_0441 [*Ruminiclostridium cellobioparum* subsp. *termitidis* CT1112]	33	43	5.50E + 00
ORF95	(73706..76168)	821	Polymerase protein [candidate division WWE3 bacterium GW2011_GWC2_44_9]	30	37	3.00E − 28
ORF96	(76155..77018)	288	Guanylate kinase [*Emticicia* sp. MM]	39	62	6.00E − 24
ORF97	(77327..78076)	250	MerR family transcriptional regulator [*Bacillus* sp. EB01]	23	54	1.50E + 00
ORF98	(78095..78961)	289	Fic family protein [*Fusobacterium nucleatum*]	38	66	8.00E − 30
ORF99	(78937..79356)	140	Hypothetical protein BpsS36_00041 [*Bacillus phage* vB_BpsS-36]	42	94	6.00E − 18
ORF100	(79396..79920)	175	Siphovirus Gp157 family protein [*Fusobacterium nucleatum*]	51	89	3.00E − 39
ORF101	(79933..80406)	158	No significant similarity found			
ORF102	(80372..80731)	120	Hypothetical protein TBLA_0I01080 [*Tetrapisispora blattae* CBS 6284]	31	66	9.50E + 00
ORF103	(80744..81175)	144	O-acetyl-ADP-ribose deacetylase 1-like [*Branchiostoma belcheri*]	44	98	3.00E − 36
ORF104	(81177..81731)	185	Hypothetical protein [*Clostridium botulinum*]	34	96	1.00E − 20
ORF105	(81819..82436)	206	Hypothetical protein [*Fusobacterium nucleatum*]	32	83	2.00E − 13
ORF106	(82601..83236)	212	Hypothetical protein [*Clostridium sp*. 12(A)]	49	85	7.00E − 53
ORF107	(83330..84430)	367	DNA cytosine methyltransferase [*Methanobrevibacter ruminantium*]	31	98	9.00E − 57
ORF108	(84452..84640)	63	No significant similarity found			
ORF109	(84691..86247)	519	Hypothetical protein FUSO4_11650 [*Fusobacterium necrophorum* DJ-1]	49	97	7.00E − 166
ORF110	(86400..86783)	128	Isovaleryl-CoA dehydrogenase [*Erythrobacter gangjinensis*]	40	50	1.30E − 01
ORF111	(86773..87030)	86	Hypothetical protein ABS80_03590 [*Pseudonocardia sp*. SCN 72–51]	33	60	6.20E − 01
ORF112	(87053..88336)	428	ATP dependent DNA ligase domain protein [*Clostridioides difficile*]	37	97	2.00E − 67
ORF113	(88329..88946)	206	VP1 protein, partial [Coxsackievirus A16]	33	23	1.70E + 00
ORF114	(88947..89186)	80	No significant similarity found			
ORF115	(89161..89643)	161	DNA repair protein MmcB-related protein [*Fusobacterium*]	30	88	1.00E − 15
ORF116	(89640..89858)	73	No significant similarity found			
ORF117	(89842..90486)	215	Hypothetical protein [*Fusobacterium necrophorum*]	45	80	1.00E − 41
ORF118	(90575..91204)	210	Hypothetical protein [*Adhaeribacter aquaticus*]	25	59	4.30E + 00
ORF119	(91309..92733)	475	Hypothetical protein [Ralstonia phage RSP15]	27	67	2.00E − 25
ORF120	(92743..93252)	170	dUTP diphosphatase [*Clostridium bartlettii* CAG:1329]	41	99	5.00E − 28
ORF121	(93249..94526)	426	Hypothetical protein [*Bacillus endophyticus*]	24	73	8.00E − 26
ORF122	(94529..95317)	263	ORF6N domain-containing protein [*Fusobacterium nucleatum*]	43	77	5.00E − 44
ORF123	(95417..96418)	334	DNA primase (bacterial type) [*Chlamydia trachomatis*]	39	50	1.00E − 18
ORF124	(96431..97912)	494	DNA helicase [*Clostridium botulinum*]	29	54	4.00E − 25
ORF125	(97909..98334)	142	epsC Polysaccharide biosynthesis protein, protein-tyrosine-phosphatase [*Lactococcus lactis* subsp. *lactis*]	33	52	1.90E + 00
ORF126	(98404..99702)	432	No significant similarity found			
ORF127	(99709..100146)	146	Phage antirepressor [*Clostridium innocuum*]	41	79	4.00E − 22
ORF128	(100272..100976)	235	ATP-binding protein [*Peptoniphilus obesi*]	37	91	2.00E − 38
ORF129	(100986..101510)	175	Hypothetical protein DLH72_04485 [*Candidatus Gracilibacteria* bacterium]	36	96	4.00E − 17
ORF130	(101497..101679)	61	No significant similarity found			
ORF131	(101885..102676)	264	DNA adenine methylase [*Ruminococcus albus*]	39	92	3.00E − 41
ORF132	(102741..103346)	202	Ribonuclease HI [*Fusobacterium russii*]	53	99	1.00E − 63
ORF133	(103346..103561)	72	No significant similarity found			
ORF134	(103545..103769)	75	Hypothetical protein K457DRAFT_16159 [*Mortierella elongata* AG-77]	40	46	6.00E + 00
ORF135	(103871..104125)	85	No significant similarity found			
ORF136	(104118..104633)	172	Hypothetical protein [*Selenomonas bovis*]	52	25	5.00E − 04
ORF137	(104773..104997)	75	Multidrug efflux RND transporter permease subunit [*Motiliproteus coralliicola*]	30	98	5.50E + 00
ORF138	(104997..105212)	72	Peptide ABC transporter substrate-binding protein [*Vibrio gangliei*]	38	61	4.80E + 00
ORF139	(105199..105858)	220	Transcriptional regulator, TetR family [*Clostridium sp*. MSTE9]	30	37	1.60E + 00
ORF140	(105871..106029)	53	Hypothetical protein L915_04856, partial [*Phytophthora parasitica*]	46	49	2.60E + 00
ORF141	(106133..106942)	270	Prohibitin family protein [*Fusobacterium periodonticum*]	77	99	3.00E − 150
ORF142	(106942..107316)	125	Hypothetical protein [*Fusobacterium necrophorum*]	48	37	1.40E + 00
ORF143	(107317..107580)	88	Type II toxin-antitoxin system HipA family toxin [*Prevotella sp*. ICM33]	29	75	5.20E + 00
ORF144	(107584..108336)	251	Hypothetical protein [*Corynebacterium matruchotii*]	52	98	2.00E − 89
ORF145	(108336..108842)	169	Hypothetical protein [*Fusobacterium nucleatum*]	50	33	1.00E − 06
ORF146	(108939..109391)	151	Furin-1 precursor [*Schistosoma japonicum*]	32	53	9.00E − 02
ORF147	(109396..109905)	170	No significant similarity found			
ORF148	(109906..110742)	279	Bifunctional methylenetetrahydrofolate dehydrogenase/methenyltetrahydrofolate cyclohydrolase [*Agrococcus casei*]	29	97	3.00E − 13
ORF149	(110730..111284)	185	Hypothetical protein A2Y22_00670 [*Clostridiales* bacterium GWD2_32_59]	27	74	5.00E + 00
ORF150	(111332..111955)	208	TPA: tRNA (adenine-N(6)-)-methyltransferase [*Candidatus Gastranaerophilales* bacterium HUM_13]	39	92	3.00E − 40
ORF151	(112023..114062)	680	AAA family ATPase [*Pseudodesulfovibrio profundus*]	28	75	4.00E − 29
ORF152	(114111..115580)	490	Hypothetical protein [*Clostridium botulinum*]	37	85	1.00E − 69
ORF153	115966..116148	61	No significant similarity found			
ORF154	116254..116772	173	No significant similarity found			
ORF155	116783..117202	140	No significant similarity found			
ORF156	117202..117636	145	Heparinase [*Paenibacillus macerans*]	33	37	4.20E + 00
ORF157	117630..117812	61	No significant similarity found			
ORF158	117915..118142	76	No significant similarity found			
ORF159	118123..119262	380	Hypothetical protein [*Fusobacterium mortiferum*]	68	31	4.00E − 40
ORF160	119268..119579	104	Glycosyltransferase [*Kingella kingae*]	35	62	7.00E + 00
ORF161	119675..119983	103	Hypothetical protein [*Streptococcus sobrinus*]	35	86	5.00E − 15
ORF162	119997..120386	130	Hypothetical protein [*Streptococcus oralis*]	32	46	3.20E − 02
ORF163	120534..120965	144	Hypothetical protein [*Fusobacterium periodonticum*]	33	84	3.00E − 10
ORF164	120975..121694	240	No significant similarity found			
ORF165	121708..122073	122	TonB-dependent receptor [*Mucilaginibacter* sp. OK098]	31	87	3.70E + 00
ORF166	122070..124286	739	Metallophosphoesterase [*Leptotrichia hofstadii*]	49	99	0.00E + 00
ORF167	124339..124518	60	No significant similarity found			
ORF168	125623..125841	73	No significant similarity found			
ORF169	125880..126119	80	Glutaredoxin [*Fusobacterium nucleatum* subsp. *nucleatum*]	56	78	8.00E − 17
ORF170	126477..126656	60	No significant similarity found			
ORF171	126614..126901	96	HU family DNA-binding protein [*Neisseria weaveri*]	44	93	5.00E − 17
ORF172	127245..128036	264	ORF6N domain-containing protein [*Fusobacterium necrophorum*]	73	46	3.00E − 58
ORF173	128097..128273	59	No significant similarity found			
ORF174	128354..129103	250	Hypothetical protein CANCADRAFT_31211 [*Tortispora caseinolytica* NRRL Y-17796]	33	23	9.00E + 00
ORF175	129197..129547	117	DUF1353 domain-containing protein [*Fusobacterium necrophorum*]	47	76	6.00E − 21
ORF176	129564..129980	139	Hypothetical protein [*Fusobacterium necrophorum*]	39	90	2.00E − 23
ORF177	129980..130537	186	N-acetylmuramoyl-L-alanine amidase [*Fusobacterium necrophorum*]	44	95	1.00E − 36
ORF178	130553..130858	102	Hypothetical protein [*Aeromonas enteropelogenes*]	40	50	1.40E + 00

**Figure 2:**
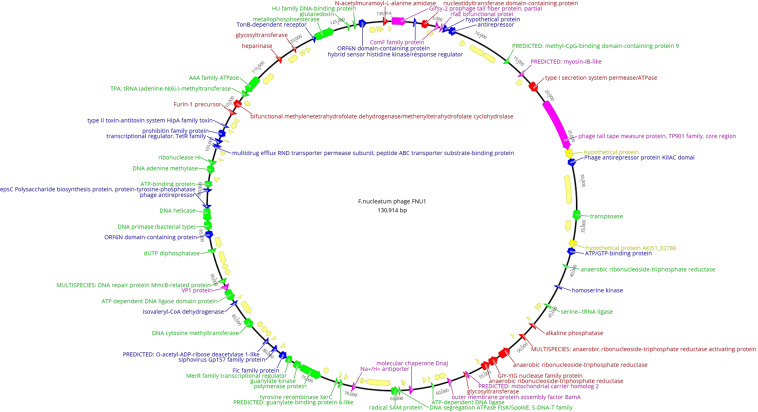
FNU1 functional genome map with putative structural genes (pink), putative DNA manipulation genes (green), putative regulatory genes (blue), putative lytic genes (red) and hypothetical genes (yellow) marked. Although majority of genes couldn’t be assigned functionality, a pattern of clustering of related genes was observed. Genes in anticlockwise orientation were comprised mostly of putative DNA manipulation genes (between 65000 and 130000 bp and possibly involved in infection & packaging), as well as cluster of putative lysis genes (between 45000 and 55000 bp).

Functional genomics predictions were mapped using the Geneious software 11.0.5 (Fig. 2) with putative structural genes (pink), putative DNA manipulation genes (green), putative regulatory genes (blue), putative lytic genes (red) and hypothetical genes (yellow) marked. Although the majority of genes couldn’t be assigned functionality, a pattern of clustering of related genes was observed (Fig. 2). Putative structural genes appeared to be orientated in a clockwise direction while the rest were orientated anticlockwise. The putative structural genes were interspaced with putative lysis and regulatory genes, as well as other genes that have no known functionality or homology located between sites 1 and 40000 bp in the genome map (Fig. 2). The genes in anticlockwise orientation were comprised mostly of putative DNA manipulation genes located between sites 65000 and 130000 bp in the genome map (Fig. 2) that may be involved in the infection and packaging processes, as well as a cluster of putative lysis genes (located between 45000 and 55000 bp).

### tRNAs and tmRNAs in the *F. nucleatum* bacteriophage FNU1 genome

There were six putative tRNAs identified using ARAGON and tRNAscan-SE 2.0. These included five that did not have any introns and one with a C-loop intron. The tRNA with a C-loop intron was a histidine-tRNA of 74 bp and GC content of 43.2%. The other five introns included an isoleucine-tRNA (91 bp, GC = 36.3%), a proline-tRNA (76 bp, GC = 47.4%), a serine-tRNA (87 bp, GC = 43.7%), a tyrosine-tRNA (89 bp, GC = 40.4%) and a cysteine-tRNA (74 bp, GC = 37.8). All introns were in a single cluster located at 44553 bp to 45210 bp in the genome (Fig. 2). No tmRNA genes were found in the FNU1 genome sequence.

### Putative bacteriophage FNU1 defence against bacterial anti-phage systems

Conserved protein family (Pfam) prediction on BLASTp analysis indicated there were 13 ORFs with predicted Pfam domains that may be involved in bacteriophage FNU1 evading host immunity (Table 2). These included at least three genes each putatively encoding antirepressors, methylation genes and toxin-antitoxin systems. According to the CRISPR (Clustered Regularly Interspaced Short Palindromic Repeats) database (http://crispr.i2bc.paris-saclay.fr/)^36^, no CRISPR regions were found in the phage FNU1 genome.

**Table 2:** Putative phage defence mechanisms against bacterial anti-phage immunity.

ORF	Coordinates	Protein family	Pfam	E- value	Function
ORF10	5884..6558	Phage regulatory protein Rha (Phage_pRha) (N-terminal)	09669	2.95E-22	Phage regulatory proteins usually found in temperate phages and bacterial prophage regions and include rha genes that interfere with bacterial infection in strains that lack integration host factor [52]
ORF10	5884..6558	ORF6C domain (C-terminal)	10552	1.14E-13	Antirepressor protein [52]
ORF11	6577..7395	Phage antirepressor protein KilAC domain	03374	9.63E-29	Antirepressor protein [52]
ORF27	27390..28244	Phage antirepressor protein KilAC domain	03374	8.61E-18	Antirepressor protein [52]
ORF34	37551..38327	AAA domain, putative AbiEii toxin	13304	7.61E-05	Type IV toxin antitoxin system that may be part of the abortive phage resistance TA system [53]
ORF82	(64738..65517)	Radical SAM superfamily	04055	2.25E-09	Radical SAM proteins catalyse different reactions from unusual methylations, isomerisation, sulphur insertion, ring formation, anaerobic oxidation and protein radical formation[54]
ORF98	(78095..78961)	Fic/DOC family	02661	5.54E-11	This family consists of the Fic (Filamentation Induced by cAMP) protein and DOC (death on curing) proteins. The Fic protein is involved in the regulation of cell division via folate metabolism. DOC will cure bacterial cells of prophage and also target protein synthesis machinery and inducing a reversible growth arrest. This arrest can be reversed by its antitoxin partner Phd (prevents host death) [55]
ORF100	(79396..79920)	Siphovirus Gp157	05565	1.79E-33	Bacteria that contain genes coding siphovirus GP157, a protein of *Streptococcus thermophiles* SFi phages are thought to have an increased resistance to phage infection[56]
ORF107	(83330..84430)	C-5 cytosine-specific DNA methylase	00145	4.45E-49	These enzymes specifically methylate the C-5 carbon of cytosines in DNA to produce C5-methylcytosine [57]
ORF127	(99709..100146)	Phage antirepressor protein KilAC domain	03374	3.69E-07	An antirepressor protein[52]
ORF131	(101885..102676)	D12 class N6 adenine-specific DNA methyltransferase	02086	5.16E-04	These enzymes will specifically methylate the amino group at the C-6 position of adenines in DNA [58]
ORF132	(102741..103346)	Caulimovirus viroplasmin (N - Terminal)	01693	3.89E-04	These form the main components of viral inclusion bodies where viral assembly, DNA synthesis and accumulation takes place [59]
ORF159	118123..119262	Phage regulatory protein Rha (Phage_pRha) (N-terminal)	09669	2.28E-04	Phage regulatory proteins that are usually found in temperate phages and bacterial prophage regions and include rha gens that interferes with bacterial infection in strains that lack integration host factor.[52]
ORF159	118123..119262	Phage antirepressor protein KilAC domain (C - Terminal)	03374	2.68E-15	Antirepressor protein [52].
ORF172	127245..128036	ORF6N domain (N-terminal)	10543	1.23E-16	Antirepressor protein [52].
ORF172	127245..128036	Phage antirepressor protein KilAC domain (C - Terminal)	03374	1.52E-18	Antirepressor protein [52].

### Phylogenetic relatedness with other bacteriophage targeting oral bacteria

**Figure 3:**
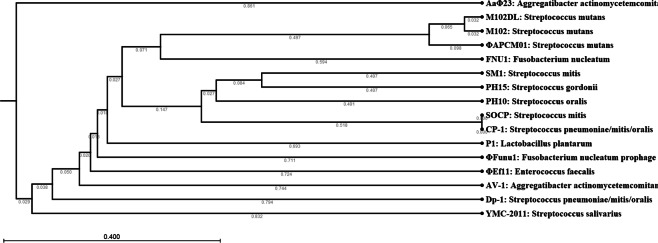
Phylogenetic analysis of FNU1 in relation to other bacteriophages associated with oral bacteria. FNU1 was most closely related to *Streptococcus mutans* and other *Streptococcu*s spp. bacteriophages.

The genome of bacteriophage FNU1 showed little homology to any bacteriophage deposited in NCBI Genbank. To understand the relatedness of FNU1 to other bacteriophages, a phylogenetic tree was constructed. Whole genomes of bacteriophages targeting oral bacteria were downloaded from NCBI Genbank and compared with FNU1 as they are found in the same microenvironment. Bacteriophage FNU1 was found to be most closely related to *Streptococcus mutans* and *Streptococcus* spp. bacteriophages (Fig. 3). The only *F. nucleatum* bacteriophage genome in the database is the prophage ΦFunu1, which is phylogenetically distant from FNU1, sharing very little genetic homology. ΦFunu1 branches off earlier in the phylogenetic tree and is most closely related to P1, a bacteriophage for *Lactobacillus plantarum* and the bacteriophage ΦEf11 for *Enterococcus faecalis* (Fig. 3).

### Effect of bacteriophage FNU1 on *F. nucleatum* biofilm mass

**Figure 4:**
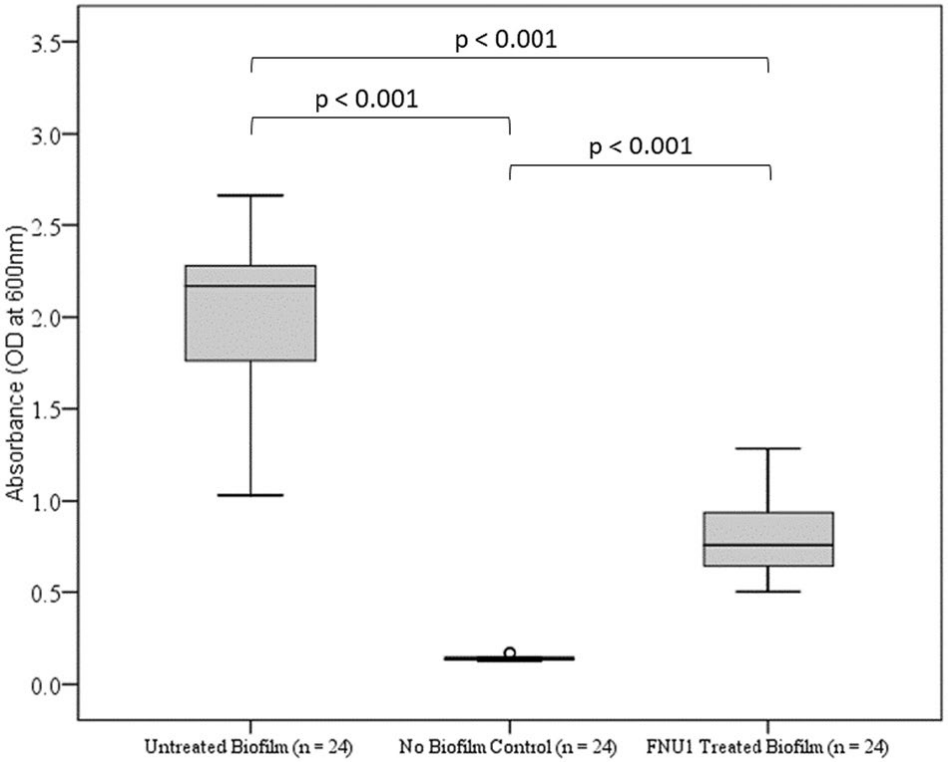
Significant reduction of *Fusobacterium nucleatum* biofilm treated with FNU1 bacteriophage.

To evaluate the potential application of bacteriophage FNU1 in the treatment of gastro-intestinal biofilms, a biofilm model of *F. nucleatum* was generated as described above. The median [Inter-Quartile Range (IQR)] absorbance at OD_600_ of the biofilm without bacteriophage treatment was 2.17 (1.81–2.21). This was significantly higher (p < 0.001) than that following FNU1 bacteriophage treatment for 24 h, where median (IQR) was 0.76 (0.71–0.89), or 35% of the untreated value (Fig. 4). The bacteriophage treated biofilm had a significantly higher absorbance (p < 0.001) compared to the negative control (no bacteria control): median (IQR) absorbance at OD_600_ of the negative control was 0.14 (0.14–0.15) (Fig. 4). Subtracting absorbance readings for the negative control (0.14) from both the treated (0.76) and untreated (2.17) biofilms results in untreated biofilms having an OD of 2.03 and treated biofilms having an OD of 0.62. Therefore, FNU1 phage treatment results in a 70% reduction in *F. nucleatum* biomass.

### Viability of the *F. nucleatum* biofilm after treatment with bacteriophage FNU1

**Figure 5:**
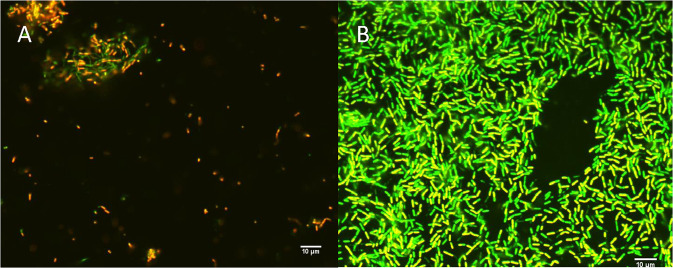
Confocal images of SYBR® gold and propidium iodide staining following FNU1 bacteriophage treated (**A**) and untreated (**B**) *Fusobacterium nucleatum* biofilm.

To evaluate the viability of *F. nucleatum* in the biofilms following treatment with bacteriophage FNU1, live/dead staining using SYBR® gold and propidium iodide was applied to biofilms formed on microscope slides and imaged using confocal microscopy. The untreated biofilm had predominantly green fluorescent cells, indicating structurally intact membranes. The bacteriophage treated biofilm showed few cells, most of which were red/yellow, indicating structurally compromised membranes, and only very few cells with intact membranes (green) in clumps (Fig. 5).

## Discussion

The novel bacteriophage FNU1 genome is over 130 kb in length and displays little homology to other known viral genomes. Because of this, it is difficult to describe any potential synteny between FNU1 and other bacteriophage genomes, although organisational similarity to some of the more abundant bacteriophages found in the human gut exists, where the structural and infection/packaging genes are in opposite orientation to each other^37^. The relatively large genome and structure of the virus, with a capsid of approximately 90 nm diameter and tail of over 300 nm in length, may have contributed to the fact that it was only able to produce clear discernible plaques when the concentration of agar it was grown on was reduced from 1.5% to 1%. The presence of several tRNAs in the FNU1 genome may indicate the requirement for additional translational mechanisms to complement those provided by the host cell. In its phylogeny, bacteriophage FNU1 clusters more closely to several bacteriophages against the oral pathogen *S. mutans*, and distantly from the only *F. nucleatum* bacteriophage in the database, the prophage ΦFunu1.

FNU1 has almost 8% of its ORFs devoted to putative defence against bacterial anti-bacteriophage systems, with several genes each coding for putative antirepressors, methylation genes to avoid restriction modification and toxin-antitoxin mechanisms that may prevent abortive infections. While some bacteriophages such as *Vibrio cholerae* ICP1 are known to carry CRISPR sequences which may contribute to their virulence^38^, the FNU1 genome had no such recognisable regions. All the *F. nucleatum* strains in the CRISPR database (http://crispr.i2bc.paris-saclay.fr/)^36^ have one CRISPR with the number of spacers ranging from 3 to 52, (average approximately 25 spacers). *Porphyromonas gingivalis*, the major etiologic agent associated with chronic periodontitis, and for which no lytic bacteriophage has yet been isolated, has very extensive CRISPR immune mechanisms^39,40^. Each *P. gingivalis* strain in the database has more than one confirmed CRISPR locus (some with a maximum of five), with the total number of spacers in each bacterial strain ranging from 14 to 136 (http://crispr.i2bc.paris-saclay.fr/). This contrasts with *C. acnes* strains, where none have a confirmed CRISPR locus, (although some are denoted as having possible CRISPR regions based on the database’s algorithms, filtering sequence length, matching repeats and amount of successive repeats)^36^, and against which there are over 80 reported bacteriophages^41^.

In this report, we demonstrate the capacity for FNU1 to disrupt established *F. nucleatum* biofilms. Our results show the capacity of FNU1 to effectively kill cells within a *F. nucleatum* biofilm, and although not as complex as polymicrobial biofilms shown to develop during periodontitis^8,29,42^, this work suggests that FNU1 has potential for application in more complex systems. *F. nucleatum* is one of the late colonisers in oral polymicrobial biofilms. Its capacity to co-aggregate intergenerically with representatives of all oral bacterial species^8^ indicates it provides the necessary scaffolding for these communities to grow, develop and flourish synergistically. We have previously shown that diverse bacteriophages are able to be formulated into dosage forms such as lozenges and pastes, and subsequently released to kill underlying bacteria *in-vitro*^43^. Both of these dosage forms would provide very useful strategies for delivery of bacteriophage such as FNU1 for testing in the treatment of periodontitis and other diseases associated with *F. nucleatum*, as they allow slower “release” of bacteriophage into the oral cavity, and in the case of toothpastes, can be used to gently massage the bacteriophage onto the tooth and gum surface.

Finally, *F. nucleatum* has recently been described as an oncobacterium, associated with a range of human cancers^4^. The organism has a causal role in tumorigenesis^12^ and also confers resistance to chemotherapy^13^. In their animal model, Bullman S. *et al*. demonstrated that treatment with metronidazole, an antibiotic that their *F. nucleatum* strains were sensitive to, reduced cancer cell proliferation and tumour growth. However, this approach of using antibiotics to kill *Fusobacterium* may be unsuitable clinically, as it has been previously demonstrated that perturbation of microbiota by antibiotics leads to reduced efficacy of chemoimmunotherapy for a range of cancers, including colorectal cancer^44,45^. On the other hand, the discovery of FNU1, with capacity to disrupt *F. nucleatum* biofilms, represents a potentially feasible means of targeted removal of this bacteria for microbiota manipulation in colorectal cancer management. In addition, while it has been suggested that bacteriophages in the gut virome may alter the microbiome such that *F. nucleatum* is able to overgrow and facilitate neoplasia in colon cells^46^, bacteriophages such as FNU1 may assist in overcoming such dysbiosis. That bacteriophages can be successfully formulated into suppositories, as we have previously shown^32^, may assist in delivery.

In conclusion, this work describes the first full genome sequence and functional characterisation of a novel lytic bacteriophage against *F. nucleatum*, a bacterium associated with periodontitis as well as cancers of the GI tract such as colon cancer. FNU1 is unique in that it shares very little homology with other known bacteriophages. Functionally, FNU1 is capable of breaking down *F. nucleatum* biofilms and lysing the bacterial cells composing the biofilm. This bacteriophage, then, is able to be tested in more complex oral biofilm assays and could potentially be tested *in-vivo* to assess capacity to treat periodontitis, as well as possibly assist in colon cancer treatment, following formulation in appropriate dosage forms.
