# Author Correction: Genomic, morphological and functional characterisation of novel bacteriophage FNU1 capable of disrupting *Fusobacterium nucleatum* biofilms

**DOI:** 10.1038/s41598-023-38412-2

**Published:** 2023-07-13

**Authors:** Mwila Kabwe, Teagan L. Brown, Stuart Dashper, Lachlan Speirs, Heng Ku, Steve Petrovski, Hiu Tat Chan, Peter Lock, Joseph Tucci

**Affiliations:** 1 Department of Pharmacy and Biomedical Sciences, La Trobe Institute for Molecular Science, La Trobe University, Victoria, Australia; 2 Melbourne Dental School, University of Melbourne, Victoria, Australia; 3 Department of Physiology, Anatomy and Microbiology, La Trobe University, Victoria, Australia; 4 Department of Microbiology, Royal Melbourne Hospital, Victoria, Australia; 5 La Trobe Institute for Molecular Science, La Trobe University, Victoria, Australia

Correction to: *Scientific Reports* https://doi.org/10.1038/s41598-019-45549-6, published online 24 June 2019

This Article contains errors.

Statement clarifying availability of the materials underlying this study has not been originally included. Materials availability section is included below.

## Data Availability

Samples of *Fusobacterium* bacteriophage FNU1 can only be shared by the authors for non-commercial purposes and under material transfer agreement. Readers wishing to obtain them should contact Latrobe University Commercialisation Office.

